# An association study of DRD2 and COMT polymorphisms with novelty seeking and harm avoidance scores, in two independent samples of depressed patients

**DOI:** 10.1186/1744-9081-3-3

**Published:** 2007-01-11

**Authors:** Katrina J Light, Peter R Joyce, Suzanne E Luty, Roger T Mulder, Janet D Carter, Christopher MA Frampton, Allison L Miller, Martin A Kennedy

**Affiliations:** 1 Department of Psychological Medicine, Christchurch School of Medicine & Health Sciences, PO Box 4345, Christchurch, New Zealand; 2 Department of Psychology, University of Canterbury, Private Bag 4800, Christchurch, New Zealand; 3 Department of Pathology, Christchurch School of Medicine & Health Sciences, PO Box 4345, Christchurch, New Zealand

## Abstract

**Background:**

It was recently reported that an interaction of the dopamine D2 receptor (DRD2) and catechol-O-methyltransferase (COMT) influences the behavioural approach system – as measured using Carver and White's Behavioural Inhibition and Behavioural Approach System (BIS/BAS) scales – in a sample of healthy German subjects. The Temperament and Character Inventory (TCI), in particular the novelty seeking (NS) and harm avoidance (HA) scales, correlates moderately with the BIS/BAS measure. This study aimed to examine support for an association of DRD2 and COMT with behavioural activation, using the TCI within two independent samples of depressed outpatients (for both samples n = 146).

**Methods:**

Two clinical samples of depressed patients were ascertained to assess the efficacy of two different pharmacotherapy and psychotherapy treatments. Analysis of variance (ANOVA) was used to analyse NS and HA scale and subscale scores with respect to gene loci within each clinical sample. Analysis of covariance were undertaken to examine the association of age and gender with NS and HA scores. An association of age group or gender with gene loci were explored using chi-squared tests, in each sample.

**Results:**

No significant effect of DRD2 or COMT, either independently or as an interaction, on NS or HA scores was observed, within either sample. Whilst age was significantly negatively associated with NS scores, including age in the two- and three-way interactions did not affect the significance of the association of personality with gene loci.

**Conclusion:**

This study suggests that the COMT-DRD2 Equilibrium Model of Positive Emotionality recently proposed by Reuter and his colleagues is not applicable amongst currently depressed individuals, whose behavioural approach and inhibition tendencies have been assessed using the TCI.

## Background

Reuter et al. [1] recently reported that the dopamine D2 receptor (*DRD2*) and catechol-O-methyltransferase (*COMT*) genes are associated with variation in individuals' behavioural approach system. The *DRD2 *TaqI A1 and *COMT *Val alleles were not individually associated with behaviour, yet an interaction of the loci significantly predicted differences in response. An individual's behavioural approach system (BAS), as well as their behavioural inhibition system (BIS), were assessed using Carver and White's [2] BIS/BAS scales. The BAS represents an individual's sensitivity to appetitive stimuli, such as reward or avoidance of punishment [2]. Meanwhile, the BIS comprises an individual's inhibition towards goals due to sensitivity to aversive stimuli or extreme novelty [2]. Reuter et al. [1] found that higher BAS scores, in particular on the 'Drive' and 'Fun-seeking' scales, were observed in those with Val-/A1- or Val+/A1+ genotype. No support was found for an effect of these gene loci on the individuals' BIS scores [1]. Gender had a significant effect on BIS scores, both as an independent variable and in association with the presence/absence of the *COMT *Met allele. Men possessing the *COMT *Met allele had lower BIS scores than men without this allele, whilst the inverse pattern occurred amongst women [1].

*DRD2 *and *COMT *have been investigated as candidate genes for behaviour motivation because of their essential roles within the dopamine system. The enzymatic activity of COMT is attenuated by the occurrence of a single nucleotide polymorphism (G>A) which causes an amino acid substitution (from a valine (Val) to a methionine (Met) residue) at codon 158 [3]. The *DRD2 *TaqIA polymorphism is a single nucleotide transition which creates a restriction fragment polymorphism [4]. The A1 allele has been associated with a 30–40% reduction in D2 dopamine receptor density, compared to individuals homozygous for the A2 allele [5-8]. Both polymorphisms have been associated with measures of behavioural motivation, including Cloninger's harm avoidance (HA) and novelty seeking (NS) scales. Enoch et al. [9] found that females homozygous for the *COMT *Met allele reported the highest HA scores. Within two samples of young males, the presence of the *DRD2 *TaqI A1 polymorphism was associated with increased NS [10,11].

Carver and White's [2] BIS/BAS scales represent the operationalisation of Jeffrey Gray's Reinforcement Sensitivity Theory. Gray and Cloninger's personality models are conceptually equivalent [2]. They both theorise that human behaviour is based on an interplay of at least two biological self-regulatory systems – one which is responsive to aversive stimuli (BIS or HA) and the other to appetitive stimuli (BAS or NS). Concordantly, positive associations have been found between HA and BIS, and NS and the BAS fun-seeking scale [2,12]. This paper reports our finding that DRD2 and COMT are not associated with behavioural activation in two independent Caucasian samples of depressed patients assessed with Cloninger's Temperament and Character Inventory (TCI) [13].

## Methods

### Patients

#### Clinical sample 1

Patients were recruited for a study examining predictors of outcome in depressed patients randomised to treatment with either fluoxetine or nortriptyline [14]. Patients had to be older than 18 years and have a principal current diagnosis of major depression. Individuals were excluded if they had a history of mania (i.e. bipolar I disorder), schizophrenia, or a severe current alcohol or drug dependence. Patients were required to have no serious physical illness and to be free of psychotropic drugs for a minimum of two weeks or five drug half lives.

Within the sample of 195 depressed patients, the mean Montgomery-Asberg [15] depression score was 30.7 (± 6.6), 60% had recurrent depression and 48% met DSM-IV criteria for melancholia. Complete genetic and personality data were available for 146 individuals, which included 142 Europeans, three Maori and one Asian. Only these 146 individuals have been included in the following analyses.

#### Clinical sample 2

This clinical sample was recruited for a study examining predictors of response to interpersonal psychotherapy (IPT) and cognitive-behavioural therapy (CBT) amongst depressed patients. Individuals were 18 years or older, with a principal current diagnosis of major depression. The exclusion criteria were the same as for clinical sample 1.

Within this sample of 177 depressed patients, the mean Montgomery- Asberg depression score was 23.8 (± 6.4), 72% had recurrent depression and 38% met DSM-IV criteria for melancholia. Only the 146 individuals for whom complete genetic and personality data was available have been included in the following analyses. The sample included 127 Europeans, eight Asians, seven Maori, one Pacific Islander and three individuals who identified as 'other'.

### Assessment

Both clinical studies were approved by the Canterbury (New Zealand) Ethics Committee. After giving informed consent, the patients attended a detailed clinical and neurobiological assessment, which included giving a blood sample for DNA extraction. The clinical assessment was conducted by a psychiatrist or clinical psychologist using the Structured Clinical Interview for DSM-IV (SCID) [16]. Ratings on the Hamilton and Montgomery-Asberg depression rating scales were made. Patients completed a series of self-report questionnaires, including the SCID Personality Questionnaire (SCID-PQ) and the Temperament and Character Inventory (TCI) [13]. After these baseline assessments, patients in clinical sample 1 were randomised to treatment with either fluoxetine or nortriptyline, and patients in clinical sample 2 were randomised to treatment with either IPT or CBT. After commencing treatment, a trained psychiatrist or clinical psychologist completed the SCID-II for DSM-IV [17] to assess the presence of axis II personality disorders.

### Laboratory methodology

The *DRD2 *TaqI A polymorphism was assayed as described by Grandy et al. [18]. The *COMT *158Val>Met variant was determined using a specifically designed one-tube allele specific PCR protocol. The G (Val) allele was detected using the primers 5'-TCACCCAGCGGATGGTGGATTTCGCTGGGG-3' and 5'-AACGTGGTGTGAACACCTGGTGGGGAG-3', yielding a 143 bp product. The A (Met) allele was detected with the primers 5'-CGGGTCAGGCATGCACACCTTGTCCTTCCT-3' and 5'-TGCTGTCACCAGGGGCGAGGCTCATCA-3', generating a 116 bp product. A 200 bp internal control band was also present. All four primers were included in a 10 μl reaction with 20–100 ng genomic DNA, HotMaster Taq (Eppendorf™), 0.2 mM each dNTP and the enzyme supplier's buffer. Thermal cycling conditions were 4 minutes at 94°C followed by 30 cycles of 94°C, 62°C, and 68°C with 30 seconds at each step. Products were resolved on 2% agarose gels. The assay was validated by DNA sequencing on a proportion of each sample.

### Statistical methods

Consistent with Reuter et al. [1] the gene loci were considered in two ways: (1) presence of an allele (*DRD2 *TaqI A1 or *COMT *Val) and (2) genotype. Analysis of variance (ANOVA) was used to analyse NS and HA scale scores with respect to gene loci, analysing the main effect of each gene loci and the two-way interaction of the loci, within each clinical sample. Effect sizes for the presence of each allele in relation to HA and NS scores, in both samples, were calculated. A separate ANOVA was used to evaluate the scores on each NS and HA subscale in relation to the presence of an allele. Analysis of covariance and Least Significant Difference post-hoc tests were undertaken to examine the association of age and gender with NS and HA scores, within the two samples. To identify the effect of more substantial changes in age, the following age groups were used: 18–29 years, 30–39 years and 40+ years. Chi-squared tests were used to examine any association of age or gender with gene loci, within the samples.

Based on the concordance of the samples with respect to ascertainment criteria and assessment method, the samples were combined. The collective sample was analysed within a univariate linear model of NS and HA scores, encompassing the main effects of gene loci, age group and gender, as well as all two-way and three-way interactions involving gene loci. To account for any differences between the two samples, "study" was included as a covariate.

## Results

### Sample characteristics

Within clinical sample 1, 40% were male and 97% were Caucasian. The mean age was 31.7 years (SD 11.4 years). 80 individuals were aged 18–29 years, 26 were 30–39 years and 40 were 40+ years. The *DRD2 *TaqI A1 and A2 allele frequencies were .22 and .78, respectively. The frequency of the *COMT *Met allele was .49 and the Val allele was .51. The mean (± SD) NS score was 51.6 (± 16.4) and HA score was 67.3 (± 20.3).

Clinical sample 2 consisted of 28% males and 89% Caucasians. The mean age was 35.0 years (SD 9.8 years). Forty seven individuals were 18–29 years, 53 were 30–39 years and 46 were 40+ years. Within this sample, the *DRD2 *TaqI A1 allele frequency was .28 and the A2 allele in .72. The *COMT *Met and Val allele frequencies were .46 and .54, respectively. The allele frequencies at both loci in each sample were in Hardy-Weinberg equilibrium. The mean (± SD) NS score was 47.8 (± 15.1) and HA score was 71.1 (± 18.6).

### Associations between personality and gene loci

There was no support for a main effect of *DRD2 *or *COMT *in either sample, irrespective of whether individuals were characterised by genotype or presence of the A1 or Val allele, on HA or NS scores. The effect sizes of the A1 and Val alleles on NS scores were 0.18 and 0.018 in sample 1. In sample 2, the effect sizes were 0.02 and 0.03, respectively. The gene loci were not related to HA scores in sample 1 (effect sizes: A1= 0.07 and COMT = 0.10) or sample 2 (effect sizes: A1 = 0.10 and COMT = 0.05). We found no significant interactions of *DRD2 *TaqI A1 allele × *COMT *Val allele for either HA or NS, within either sample (Table 1). Furthermore, there was no significant effect of gene loci on any HA or NS subscale score.

**Table 1:** Mean (+ SD) novelty seeking and harm avoidance scores dependent on an individual's COMT and DRD2 genotype

DRD2	COMT	N	Mean (± SD)
**Clinical Sample 1**			
*Novelty seeking*			
A1 absent	Val absent	19	56.2 ± 14.0
	Val present	69	51.8 ± 18.3
A1 present	Val absent	19	47.4 ± 13.6
	Val present	39	51.0 ± 15.1
*DRD2 main effect*			F _(1,142) _= 2.38, p = 0.125
*COMT main effect*			F _(1,142) _= 0.015, p = 0.901
*DRD2 × COMT*			F _(1,142) _= 1.62, p = 0.206
*Harm avoidance*			
A1 absent	Val absent	19	70.8 ± 15.9
	Val present	69	67.0 ± 21.0
A1 present	Val absent	19	66.9 ± 23.4
	Val present	39	66.3 ± 19.8
*DRD2 main effect*			F _(1,142) _= 0.356, p = 0.552
*COMT main effect*			F _(1,142) _= 0.319, p = 0.573
*DRD2 × COMT*			F _(1,142) _= 0.165, p = 0.685
**Clinical Sample 2**			
*Novelty seeking*			
A1 absent	Val absent	16	48.3 ± 18.5
	Val present	62	47.9 ± 15.9
A1 present	Val absent	14	46.4 ± 12.5
	Val present	54	47.9 ± 14.0
*DRD2 main effect*			F _(1,142) _= 0.087, p = 0.769
*COMT main effect*			F _(1,142) _= 0.027, p = 0.870
*DRD2 × COMT*			F _(1,142) _= 0.088, p = 0.767
*Harm avoidance*			
A1 absent	Val absent	16	70.7 ± 18.1
	Val present	62	72.3 ± 15.9
A1 present	Val absent	14	73.1 ± 17.5
	Val present	54	69.3 ± 22.1
*DRD2 main effect*			F _(1,142) _= 0.008, p = 0.928
*COMT main effect*			F _(1,142) _= 0.082, p = 0.775
*DRD2 × COMT*			F _(1,142) _= 0.489, p = 0.485

### Association of gender and age with personality and gene loci

There was no significant association of gender with NS and HA scores within either sample. Age was significantly associated with NS, but not HA, scores within sample 1 (F(2, 143) = 13.61, p = <0.001) and sample 2 (F(2,143) = 7.06, p = 0.001). Within sample 1, individuals aged 18–29 years had significantly higher NS scores than those aged over 30 years. Within sample 2, individuals who were younger than 39 years scored significantly higher than those who were 40+ years.

No significant association of gender with genotype or allele frequency of *DRD2 *and *COMT *was observed in either sample. Similarly, there was no significant association of age and *DRD2 *or *COMT*.

### Association of personality with gene loci, age and gender, within a combined sample

We tested all possible two-way interactions (gene loci × age/gender) and three-way interactions (*DRD2 *× *COMT *× age/gender), with respect to NS and HA total scale scores, in the combined sample and found no significant association.

## Discussion

We found no support for an association of *DRD2 *TaqI A and *COMT *Val polymorphisms with an individual's behavioural approach response, using the TCI NS scale in two samples of depressed patients. The effect size associated with the presence of either the A1 or Val allele on NS scores was very small (<0.18) providing strong evidence that the lack of association was not the result of inadequate statistical power. This contrasts with Reuter et al.'s [1] findings that these genes epistatically influence fun-seeking scores – which are correlated with TCI NS scores. Consistent with our findings, Enoch et al. [9] found that the *COMT *Val polymorphism was not associated with NS in either a Caucasian or an American Indian community sample. In contrast, Tsai et al. [19] in a sample of Chinese female nursing students found that individuals homozygous for the Val allele had higher NS [19]. Noble et al. [10] reported a significant association of NS with the *DRD2 *TaqI A polymorphism and *DRD2 *haplotype (TaqI A, TaqI B and intron 6 polymorphisms), although the association was significant for only one NS subscale. Conversely, Lee et al. [20] found no association between these three *DRD2 *polymorphisms and NS or HA scores, within a young Korean sample. Investigating two other *DRD2 *polymorphisms – Ser311Cys and -141Ins/del – within an Austrian and Japanese sample, Gebhardt et al. [21] and Katsuragi et al. [22], respectively, found no support for an association with either NS or HA.

We found no association of *DRD2 *and *COMT *polymorphisms with individuals' behavioural inhibition – based on TCI HA scores – consistent with Reuter et al. [1]. Similarly, Noble et al. [10] found that *DRD2*, examined either as the TaqI A allele or as a haplotype, was not associated with HA. These findings do not discount the possibility that these genes are at some stage involved in the development of this self-regulatory system, but rather suggest that their effect is moderated by other variables. For example, Enoch et al. [9] found that *COMT *was only associated with HA amongst female but not male subjects.

Within our samples, gender did not significantly effect behavioural inhibition scores – as found by Reuter et al. [1]. However, we found support for the finding that NS declines with age [23,24], whereby individuals aged 18–29 years scored consistently higher than individuals 40+ years. Although, in the two-way and three-way interaction, age did not alter the significance of the gene loci effects.

Two evident sources of difference between our investigation and that of the original study are the personality measure used and the sample composition. Reuter et al. [1] found a significant association with two of the three BAS subscales. One of these scales 'fun seeking' correlates moderately with NS (r =.51, p <.001), whilst the other 'drive' does not [2]. Therefore, the BIS/BAS scales evaluate behavioural approach tendencies from a slightly different perspective to that of the TCI. Reuter et al. [1] examined healthy subjects, whilst all our participants had a current major depressive disorder. A genetic association may be more evident in a community sample which includes a range of behavioural approach and inhibition responses, whilst our samples of depressed patients may be heavily weighted for high HA [24,25]. Also, as our sample is not primarily of German origin, it differs from Reuter et al.'s [1] with respect to ethnicity and therefore genetic heterogeneity may partly be responsible for the different results.

Variable findings may also reflect a lack of a direct effect of a polymorphism on the function of the gene product. For example, the functional effects associated with the *DRD2 *TaqI A polymorphism may be due to the polymorphism being located within the regulatory region of the ankyrin repeat and kinase domain containing 1 (*ANKK1*) gene [26] or being in linkage disequilibrium with another *DRD2 *variant. This indistinctness may be further compounded by grouping A1A1 carriers with A1A2 carriers (due to the low frequency of the A1 allele) for analysis. Whether this is an accurate representation of the functionality of the polymorphism remains to be determined.

## Conclusion

This study suggests that the association found by Reuter et al. [1] does not hold in a sample of depressed patients assessed using the TCI. Although, due to the interplay of metabolising enzymes and receptors it is expected that at some level these genetic factors are interdependent, yet how these facets might interact to influence personality remains unknown. Appraisal of both positive and negative results will hopefully provide some clues as to the contexts in which associations exist between genetic factors and specifically-defined behaviours.

## Competing interests

The author(s) declare that they have no competing interests.

## Authors' contributions

KL performed the statistical analyses and drafted the manuscript. PJ contributed to the conception of the study, data acquisition and assisted in the editing of the manuscript. SL, RM and JC contributed to the conception of the study, patient assessment and administration of the treatment. CF assisted with the analysis and interpretation of the statistical data. AM and MK carried out the genotyping. All authors read, provided comments and approved the final manuscript.
